# Molecular characterization and expression of *TGFβRI* and *TGFβRII* and its association with litter size in Tibetan sheep

**DOI:** 10.1002/vms3.1013

**Published:** 2023-01-07

**Authors:** Junxia Zhang, Mingming Li, Ruizhe Sun, Na He, Xiaocheng Wen, Xueping Han, Zenghai Luo

**Affiliations:** ^1^ College of Agriculture and Animal Husbandry Key Laboratory of Livestock and Poultry Genetics and Breeding on the Qinghai‐Tibet Plateau Ministry of Agriculture and Rural Affairs Plateau Livestock Genetic Resources Protection and Innovative Utilization Key Laboratory of Qinghai Province Qinghai University Xining P. R. China; ^2^ Technology Extension Service of Animal Husbandry of Qinghai Xining China

**Keywords:** genetic characteristics, TGFβRI and TGFβRII genes, Tibetan sheep

## Abstract

**Backgrounds:**

Transforming growth factor‐β (TGF‐β) type I receptor (TGFβRI) and type II receptor (TGFβRII) are the members of the TGFβ superfamily, which are potent regulators of cell proliferation and differentiation in many organ systems, and they play key roles in multiple aspects of follicle development.

**Objectives:**

We aimed to explore the characterization, expression analysis of *TGFβRI* and *TGFβRII* genes, and the association with litter size in Tibetan sheep.

**Methods:**

In this study, we cloned the complete coding sequences of *TGFβRI* and *TGFβRII* genes in Tibetan sheep and analyzed their genomic structures.

**Results:**

The results showed that percentages of sequences homology of the two proteins in Tibetan sheep were the most similar to *Ovis aries* (100%), followed by *Bos mutus* (99%). The RT‐qPCR showed that two genes were expressed widely in the different tissues of Tibetan sheep. The *TGFβRI* expression was the highest in the lung (*p* < 0.05), followed by the spleen and ovary (*p* < 0.05). The *TGFβRII* expression was significantly higher in uterus than that in lung and ovary (*p* < 0.05). In addition, the *χ* ^2^ test indicated that all ewes in the population were in Hardy–Weinberg equilibrium, and the population was in medium or low polymorphic information content status. We also found four Single Nucleotide Polymorphism (SNPs), g.9414A > G, g.28881A > G, g.28809T > C, g.10429G > A in sheep *TGFβRI* gene and g.63940C > T, g.63976C > T, g.64538C > T, g.64504T > A in *TGFβRII* gene. Three genotypes, except for g.64504T > A, and three haplotypes were identified in each gene. linkage disequilibrium analysis indicated that there was strong linkage disequilibrium in each gene. The association analysis showed that the four SNPs of *TGFβRI* were associated with litter size (*p* < 0.05), and g.63940C > T of *TGFβR*II was confirmed to be associated with litter size (*p* < 0.05).

**Conclusions:**

Based on these preliminary results, we can assume that TGFβ receptors (TGFβRI and TGFβRII) may play an important role in sheep reproduction.

## Graphical abstract

This study was undertaken to explore the characterization, expression analysis of *TGFβR I* and *TGFβR II* genes, and its association with litter size in Tibetan sheep. The TGFβ receptors (TGFβRI and TGFβR II) may play an important role in sheep reproduction.

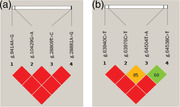

## 1 INTRODUCTION

The TGFβ superfamily is a large and expanding group of regulatory polypeptides (Kumari et al., 2021). The molecular signalling pathway of the TGFβ superfamily has been conserved throughout the six hundred million years of metazoan evolution (Loveland & Hime, 2005), which is critical for regulating a variety of developmental events, including cell proliferation, differentiation, and matrix secretion (Elvin et al., 2000; Nong et al., 2019). The family members of the TGFβ superfamily are candidates for mediating important oocyte activity (Elvin et al., 2000; Lankford & Weber, 2010). TGFβ receptor type I (TGFβRI) and the TGFβ receptor typeII (TGFβRII) are important members of the TGFβ superfamily. TGFβ signalling, important in ovary development is mediated through TGFβRI and TGFβRII. These receptors are interdependent components of a heteromeric complex, as receptor I requires receptor II for TGFβ binding and receptor II requires receptor I for signalling (Attisano & Wrana, 2002; Knight & Glister, 2003; Sun et al., 2008). TGFβ ligands bind and activate TGFβ receptor complex composed of the type II (TGFβRII) and type I subunits (TGFβRI), which phosphorylate Smad2 and Smad3. Activated Smad2/3 forms transcriptional complexes with Smad4 and other transcriptional factors and regulates the transcription of genes (Serizawa et al., 2013). It has been reported that they play an important role in many aspects of follicular development, including activation of resting primordial follicles, proliferation and apoptosis of Granulosa cells and membrane cells, steroid formation, gonadotropin receptor expression, oocyte maturation, ovulation, and luteinization (Elvin et al., 2000). The various type I and type II receptors through which each of these ligands can signal are expressed by pre‐granulosa cells/granulosa cells of the corresponding early follicle stages, making these cells potential targets for paracrine signalling (Shimasaki et al., 2004).

A few genes of the TGFβ superfamily were investigated, and their association with reproductive performance has been observed in lines of sheep (Elvin et al., 2000; Jia et al., 2020; Shi et al., 2021; Shimasaki et al., 2004; Xu et al., 2010). However, little is known about the roles of other members of the TGF‐β superfamily in Tibetan sheep; thus, the potential interaction of members of the TGFβ superfamily and their relationship with lambing traits is unclear. Therefore, the objectives of this study were to characterize the complete or partial cDNA sequences of *TGFβI* and *TGFβII*, determine the expressing mRNA encoding *TGFβRI* and *TGFβRII*, and analyze the effects of *TGFβI* and *TGFβII* on litter size in Tibetan sheep.

## 2 MATERIALS AND METHODS

### 2.1 Animals

Tibetan sheep were obtained from sheep farm (Xiangkemeiduo Sheep Industry Co. Ltd., Qinghai, China), and the experimental group included 433 ewes, which were selected randomly. The health and reproduction records of the animals were kept by the farmers. Their litter size was obtained from reproduction records. All efforts were made to minimize discomfort during the blood collection. Blood samples were collected from the jugular vein under the supervision of qualified veterinarians. Genomic DNA was extracted from blood sample of each sheep using an EasyPure Blood Genomic DNA Kit (TransGen Biotech, Beijing, China). Three ewes were selected from purebred herds of the same farm in Qinghai province. The three selected ewe (6 months old) were healthy, similar in weight, and pastured in similar conditions of grassland. After slaughtered, and the tissues from hypothalamus, hypophysis, heart, liver, spleen, lung, kidney, ovary, oviduct, uterus, rumen, duodenum, and longissimus dorsi were collected and immediately frozen in liquid nitrogen, and then stored at −80°C. The RNA of tissues was extracted by TransZol (TransGen Biotech). Total RNA for each tissue was reverse‐transcribed to cDNA by TransScript One‐Step gDNA Removal.

### 2.2 cDNA cloning and sequence analysis

The cDNA sequences of sheep *TGFβRI* and *TGFβRII* (GenBank Accession No. NM_001009224.1, XM_012179698.3, respectively) were used as templates. The primer pairs were designed using the coding regions of the two genes (Table 1). The PCR program was as follows: 94°C for 5 min; 30 cycles of 94°C for 30 s, T_m_°C for 30 s and 72°C for 40 s, followed by one cycle at 72°C for 5 min. The above PCR products were electrophoresed on a 1% agarose gel.

**TABLE 1:** Primer information and PCR conditions used in this study

Gene name	Primer name	Primer sequences (5ʹ–3ʹ)	Size (bp)	*T_m_* (°C)
*TGFβRI*	TGFβRI‐CDS‐S TGFβRI‐CDS‐A	GAGGCGAGGCTTGTTGAG TGGCAGTTTCCTGGGTCT	1751	55
*TGFβRII*	TGFβRII‐CDS1‐S TGFβRII‐CDS1‐A	GCACGTTCCCAAGTCGGTT ATGTCCTTCTCCGTCTTCC	801	61
	TGFβRII‐CDS2‐S TGFβRII‐CDS2‐A	GCTGGTCATCTTCCAAGTGACA ACCTCTTTCCACTAGTATGGCTG	1537	60
*TGFβRI*	TGFβRI‐expression‐S TGFβRI‐expression‐A	TGGCAGAGCTGTGAAGCCTTG AGCCTAGCTGCTCCATTGGCAT	77	63
*TGFβRII*	TGFβRII‐expression‐S TGFβRII‐expression‐A	CTGGCCAACAGTGGGCAGGTG CGTCTGCTTGAAGGACTCGACATT	99	63
*GAPDH*	GAPDH‐expression‐S GAPDH‐expression‐A	GCGAGATCCTGCCAACATCAAGT CCCTTCAGGTGAGCCCCAGC	105	63

The PCR product was purified using agarose gel DNA extraction kit (Takara, Dalian, China), and cloned into pMD19‐T vector (volume of 10 μl of 50 ng DNA, 50 ng pMD19‐T vector, 5 μl Solution I, incubated at 4°C overnight), then transformed into *Escherichia coli* DH5a (Takara) competent cell and grown in Luria‐Bertani (LB) agar plate with Amp. White colonies were selected (10 colonies for each sample) and cultured in liquid medium for 5 h, and then sequenced by Shanghai Sangon Biological Engineering Company. Alignments of multiple sequences were carried out with BLAST (NCBI, http://blast.ncbi.nlm.nih.gov/Blast.cgi). Open reading frame (ORF) Finder (http://www.ncbi.nlm.nih.gov/projects/gorf/) was used to determine the ORF and predict the amino acid sequence. ProtParam (http://web.expasy.org/protparam/) was used to predict the physical parameters of each protein. The hydrophilicity and hydrophobicity were analyzed using ProtScale (https://web.expasy.org/protscale/). Prediction of the secondary structure of each protein and its variants was analyzed using SOPMA (http://www.compbio.dundee.ac.uk/www‐jpred/). SWISS‐MODEL (http://swissmodel.expasy.org/) was used to predict the protein signal peptide and protein tertiary structure. Amino acid sequences alignments were conducted using DNAStar Lasergene (MegAlign), and a phylogenetic tree was established using the MEGA7 software.

### 2.3 Tissue expression analysis of sheep *TGFβRI* and *TGFβRII*

The primers for real‐time PCR were designed according to mRNA sequences of *TGFβRI* and *TGFβRII* gene (GenBank accession No: XM_004004226.4 and XM_012099309.2) (Table 1). The reaction volume was 20 μl containing 10 μl of SYBR Premix ExTaq II, 0.4 μl (10 μmol/L) forward primer, 0.4 μl (10 μmol/L) reverse primer, 1 μl cDNA, and 8.2 μl ddH_2_O. The PCR cycle consisted of 94°C for 2 min; then, 45 cycles of 94°C for 10 s, 60°C for 20 s, and 72°C for 1 s; and an extension of 72°C for 5 min. The qPCR was performed using a CFX96 Touch Real‐Time PCR (BIO‐RAD, USA). All experiments were performed in triplicate, and *GAPDH* was used as the reference gene. The 2^−∆∆CT^ method was used to analyze the data (Livak & Schmittgen, 2001).

### 2.4 SNP identification and genotyping

*TGFβRI* and *TGFβRII* genes Single Nucleotide Polymorphism (SNPs) were screened Using the dbSNP database (http://www.ncbi.nlm.nih.gov/snp) and verified by DNA sequencing. Improved multiplex ligation detection reaction (iMLDR^TM^) was used for genotyping following the instrument operating guidelines. Genotypic, allelic frequencies, and genetic parameters were directly calculated following previous description (Zhao et al., 2013). The linkage disequilibrium (LD) was conducted using the Haploview software.

### 2.5 Association analysis

The association analysis between genotypes and litter size of ewes was determined according to a general linear model (GLM) program. All statistical analyses were performed using SPSS 23.0. Results with *p* < 0.05 were considered significantly different. Based on the characteristics of sheep, the statistical model was as follows:

$$y_{ijn}=\mu +P_{i}+G_{j}+I_{PG}+e_{ijn},$$

where *y_ijn_* is the phenotypic value, *μ* is the population mean, *Pi* is the fixed effect of the *i*th parity (*i* = 1, 2, or 3), *Gj* is the fixed effect of the *j*th genotype (*j* = 1, 2, 3), *I_PG_* is the interaction effect of parity and genotype, and *e_ijn_* is the random residual.

## 3 RESULTS

### 3.1 Molecular cloning and sequence analysis of sheep *TGFβRI* and *TGFβRII*

**FIGURE 2:**
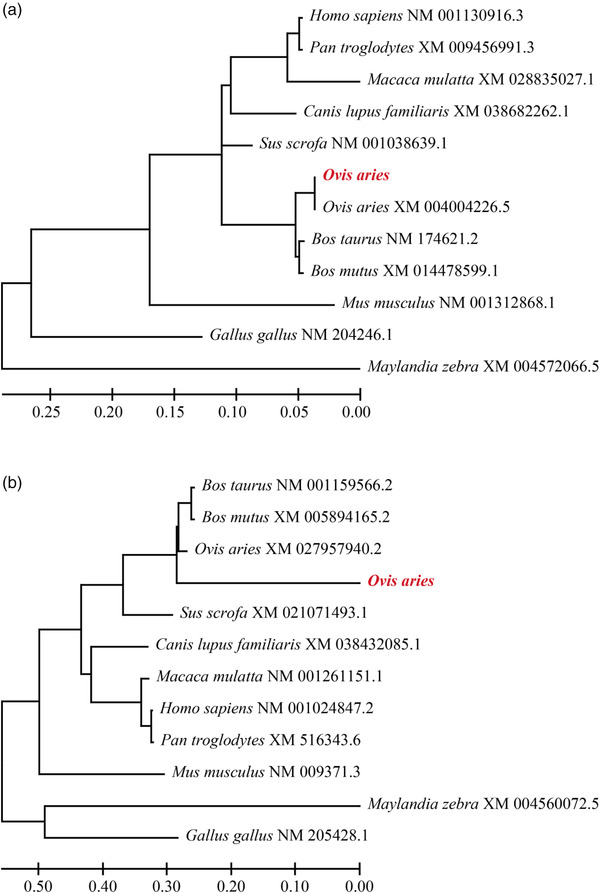
Percentage of sequences homology of *TGFβRI* (a) and *TGFβRII* (b)

In this study, 1751 bp of the sheep *TGFβRI* gene was cloned, which contained a calculated ORF of 1506 bp encoding a protein of 501 amino acid residues. Additionally, sheep TGFβRII contains ORFs of 1416 bp, and they encode proteins of 471 amino acid residues. The molecular weights of TGFβRI and TGFβRII are 55960.70 and 52879.55 Da, respectively, and the theoretical isoelectric points are 7.19 and 5.84, respectively. All of them include 20 types of amino acid composition. The total numbers of negatively charged residues (Asp + Glu) are 56 and 60, respectively, and the total numbers of positively charged residues (Arg + Lys) are 56 and 49, respectively. TGFβRI formula is C_2470_H_3936_N_688_O_723_S_35_. The total number of atoms is 7852. The Aliphatic index is 89.92; the grand average of hydropathicity (GRAVY) is −0.097; TGFβRII formula is C_2339_H_3689_N_637_O_703_S_28_. The total number of atoms is 7396. The Aliphatic index is 90.45; grand average of hydropathicity (GRAVY) is −0.170. A positive value indicates that the protein is hydrophobic, and a negative value indicates that it is hydrophilic, so all of them are hydrophilic. Subcellular localization of TGFβRI is 55.6% in endoplasmic reticulum; it is 22.2% in Golgi, 11.1% in plasma membrane, 11.1% in extracellular, including cell wall. And TGFβRII is 34.8% in nuclear; it is 26.1% in cytoplasmic, 21.7% in mitochondrial, 4.3% in endoplasmic reticulum, 4.3% in peroxisomal, 4.3% in vesicles of secretory system, 4.3% in vacuolar. The proteins of TGFβRI and TGFβRII have signal peptides. WEBSEQUENCE Number of predicted Transmembrane Helices is 2 and 1. There were potential N‐glycosylation sites at amino acids 41, 148, 268, and 348. The potential values were 0.7000, 0.8358, 0.6267, and 0.4613 in Tibetan sheep TGFβRI. There were potential N‐glycosylation sites at amino acids 70, 94, and 266. The potential values were 0.5869, 0.6930, and 0.6757 in Tibetan sheep TGFβRII. There are 50 and 45 potential phosphate sites in sheep TGFβRI and TGFβRII, respectively. Amino acid sequence alignment and percentage of sequences homology of the two proteins in *Ovis aries, Bos taurus, Bos mutus, Homo sapiens, Sus scrofa, Mus musculus, Maylandia zebra, Canis lupus familiaris, Pan troglodytes, Macaca mulatta*, and *Gallus gallus* showed that Tibetan sheep TGFβRI and TGFβRII are most similar to *O. aries* (100%), then *B. mutus* (99%), and least similar to *C. lupus familiaris* (82%), respectively (Figures 1 and 2).

FIGURE 1Amino acid sequence alignment of TGFβRI (a) and TGFβRII (b) of Tibetan sheep, sheep, cattle, yak, house mouse, human, chimpanzee, rhesus monkey, pig, dog, chicken and zebra with that of Tibetan sheep

**Figure:**
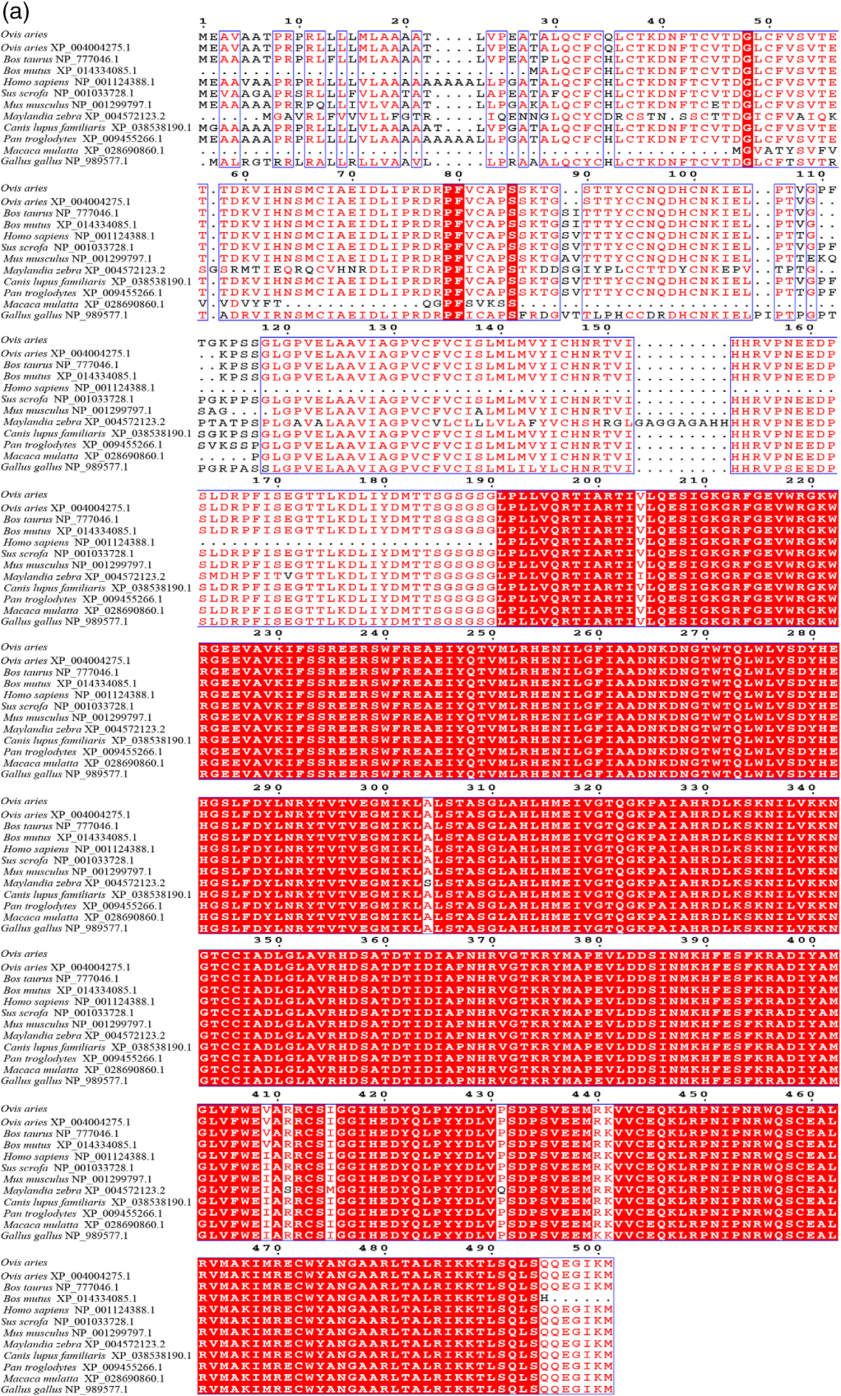

**Figure:**
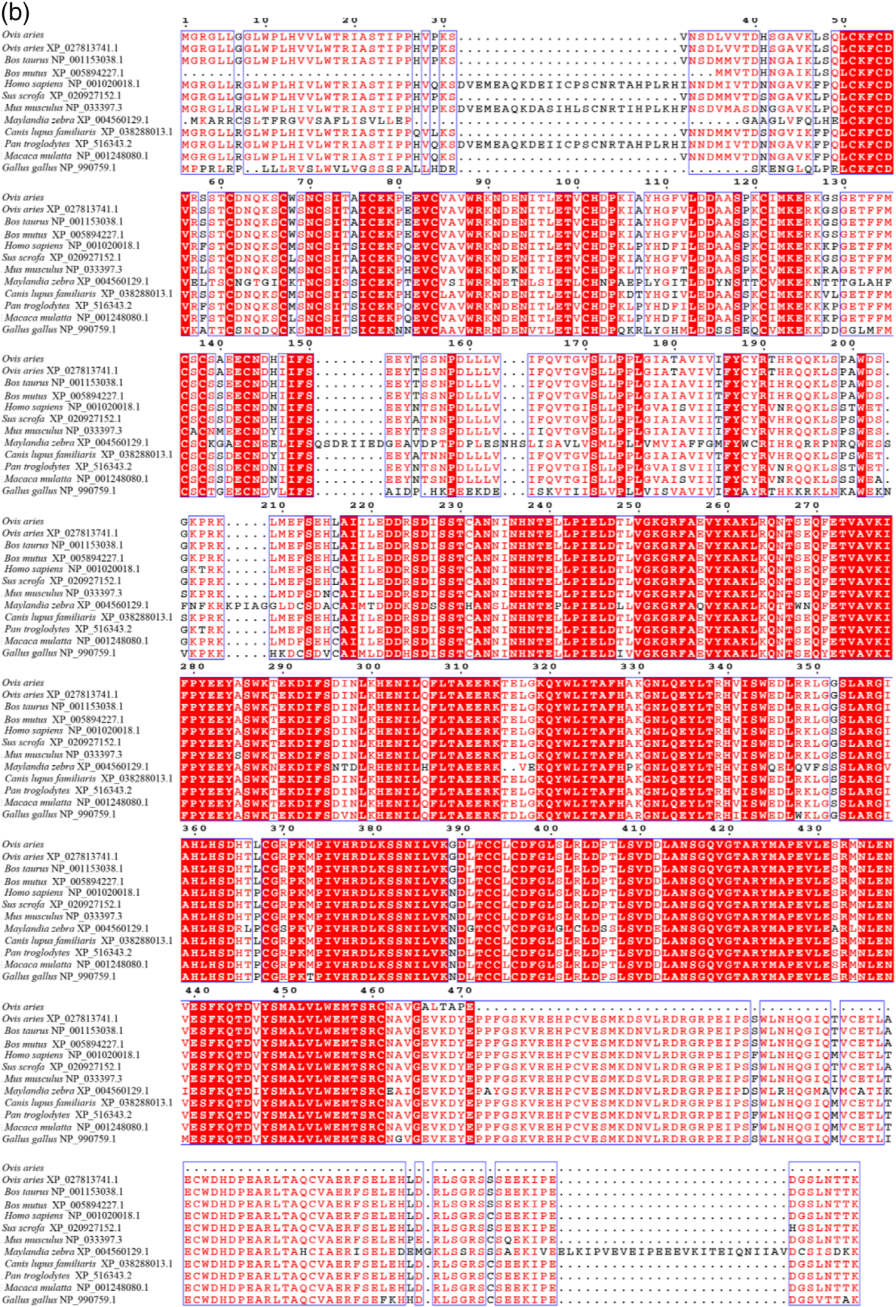

**FIGURE 3:**
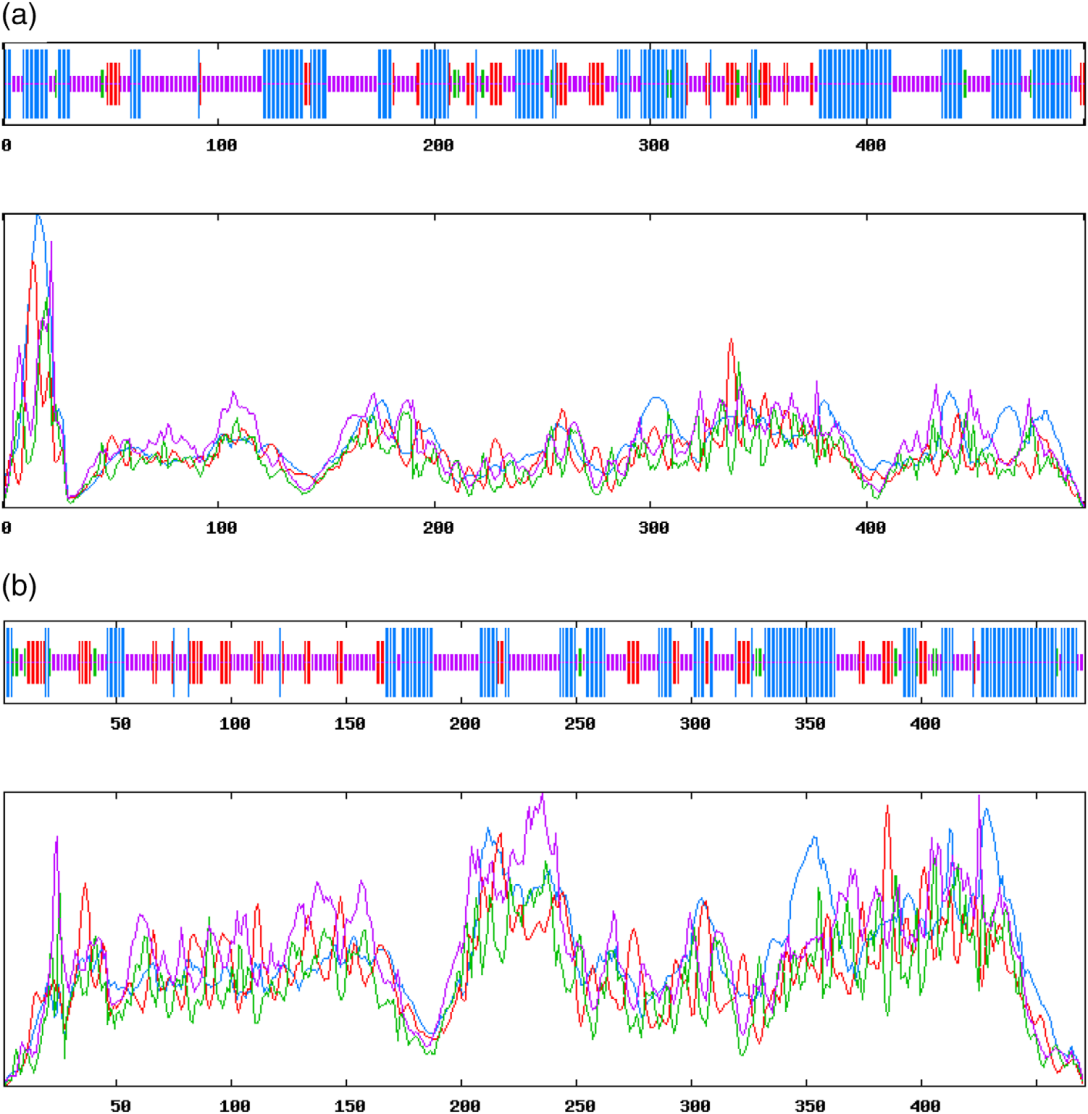
Secondary structure of TGFβRI (a) and TGFβRII (b) protein. Blue represents alpha helix, green represents beta turn, purple represents random coil, and red represents extended strand.

**FIGURE 4:**
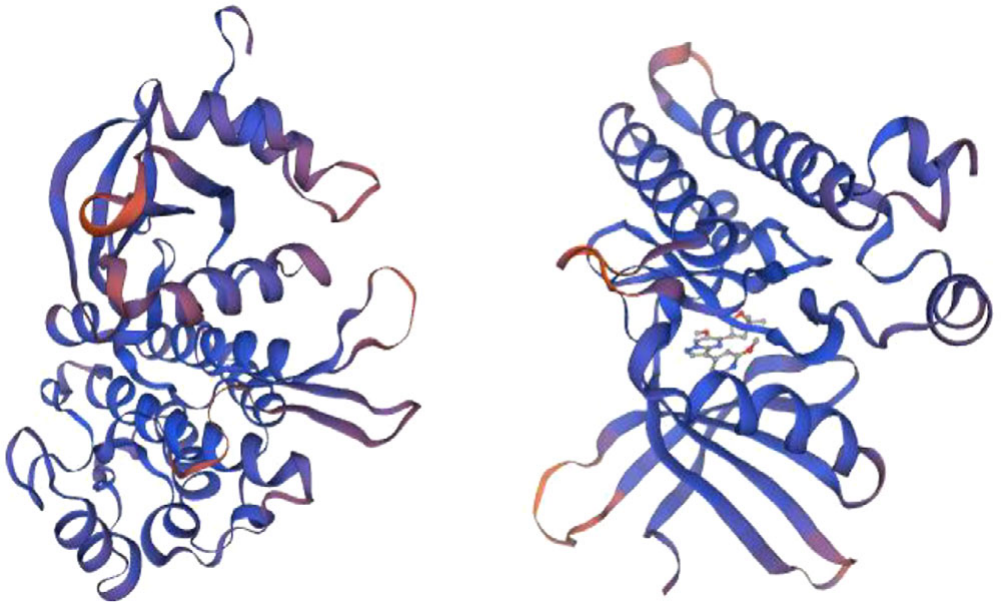
Structural features of *TGFβRI* (a) and *TGFβRII* (b)

The structure prediction of sheep TGFβRI protein was performed by online protein analysis system SOPMA. The results showed that the extension chain composed of alpha‐helix, extended strand, beta turn, and random coil accounted for 39.32%, 11.38%, 3.39%, and 67.27%, respectively, and for TGFβRII protein, they were 33.76%, 15.92%, 3.82% and 46.50%, respectively (Figures 3 and 4).

### 3.2 Expression profile analysis

**FIGURE 5:**
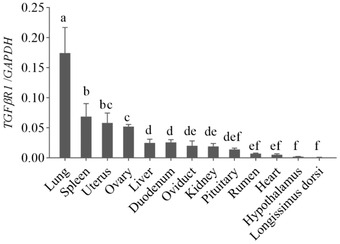
Expression of *TGFßRI* mRNA in different tissues of Tibetan sheep. *Note*: different superscripts indicate significant difference (*p* < 0.05).

**FIGURE 6:**
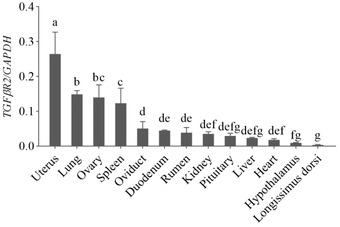
Expression of *TGFßRII* mRNA in different tissues of Tibetan sheep. *Note*: different superscripts indicate significant difference (*p* < 0.05).

The RT‐qPCR was used to investigate the general tissue distributions of *TGFβRI* and *TGFβRII*. As shown in Figures 5 and 6, two genes were widely expressed in hypothalamus, pituitary, heart, liver, spleen, lung, kidney, ovary, oviduct, uterus, rumen, duodenum, and longissimus dorsi in Tibetan sheep. The *TGFβRI* was expressed with the highest level in the lung (*p* < 0.05), followed by the spleen, uterus and ovary (*p* < 0.05), and almost no expression in longissimus dorsi. The *TGFβRII* expression was the highest in uterus than in other tissues (*p* < 0.05), followed by lung, ovary, and spleen (*p* < 0.05). There were no significant differences among oviduct, duodenum, rumen, kidney, pituitary, liver, and heart (*p* > 0.05). Except for the hypothalamus, the expression of *TGFβRII* gene in longissimus dorsi was lower than that in the other tissues (*p* < 0.05).

### 3.3 Population genetic analysis of polymorphism in sheep *TGFβRI* and *TGFβRII*

**FIGURE 7:**
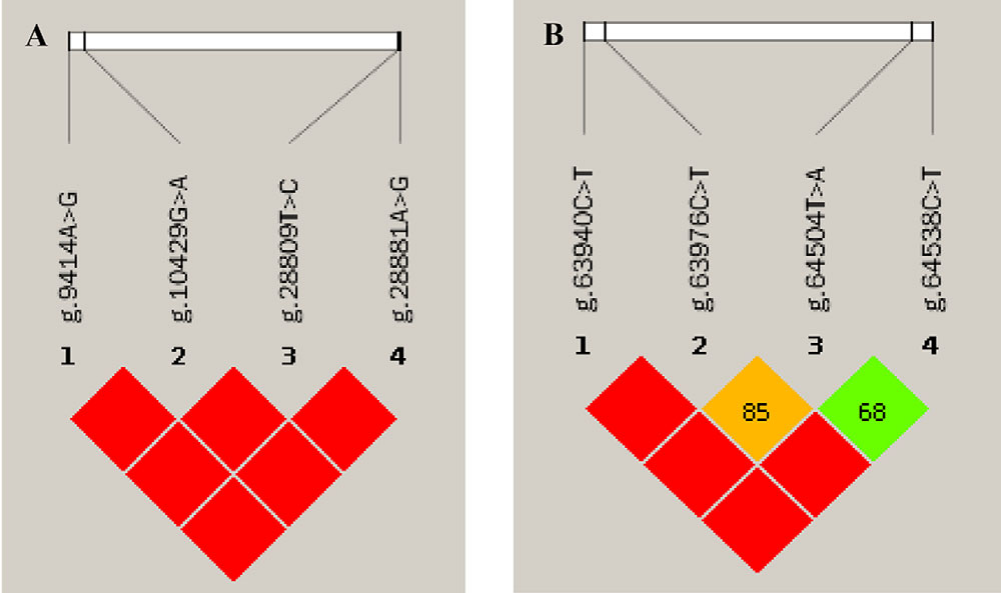
Linkage disequilibrium (LD) analysis of four SNPs of *TGFβRI* (a) and *TGFβRII* (b) gene. The number in each cell represents pairwise *r* ^2^ values (%), and *r* ^2^ value represents the correlation coefficient between the two loci.

In this study, four polymorphic nucleotide sites (SNPs) were identified in Tibetan sheep *TGFβRI* and *TGFβRII* genes, respectively. All mutations were synonymous mutations. Except for SNP g.64504T > A, the other SNPs were classified as three genotypes (Table 2), and three haplotypes were identified in each gene (Table 3). Linkage disequilibrium (*r* ^2^) block indicated strong linkage disequilibrium in two genes, respectively (Figure 7). In addition, *Ho*, *He*, *Ne*, and polymorphic information content (PIC) of Tibetan sheep *TGFβRI* were 0.72, 0.28, 1.40, and 0.24, respectively, and for *TGFβRII*, 0.76, 0.24, 1.31, and 0.21, respectively. Tibetan sheep were in medium PIC status at g.63940C > T and g.28809T > C sites, and the others have low PIC status (Table 4). The *χ* ^2^ test indicated that all ewes in the populations were in Hardy–Weinberg equilibrium.

**TABLE 2:** The frequencies of genotype and gene of SNPs (Single Nucleotide Polymorphism) sites of *TGFβR1* and *TGFβRII*

					Genotypic frequencies					
Gene	Position	CHR	Ref allele	Alt allele	R	H	D	Ref allele frequencies	Alt allele frequencies	HWE case
*TGFβRI*	g.9414A > G	2	A	G	0.88 (383)	0.11 (45)	0.01 (5)	0.94	0.06	0.07
	g.28881A > G	2	A	G	0.88 (383)	0.11 (45)	0.01 (5)	0.94	0.06	0.07
	g.28809T > C	2	T	C	0.36 (154)	0.50 (217)	0.14 (62)	0.61	0.39	0.31
	g.10429G > A	2	G	A	0.89 (356)	0.10 (44)	0.01 (3)	0.94	0.06	0.06
*TGFβRII*	g.63940C > T	19	C	T	0.40 (175)	0.44 (188)	0.16 (70)	0.62	0.38	0.13
	g.63976C > T	19	C	T	0.78 (336)	0.21 (90)	0.02 (7)	0.88	0.12	0.65
	g.64538C > T	19	C	T	0.94 (402)	0.06 (29)	– (2)	0.96	0.04	0.12
	g.64504T > A	19	T	A	0.91 (405)	0.09 (38)	– (0)	0.96	0.04	1.00

Abbreviations: CHR, chromosome; Ref allele, reference allele; Alt allele, the other allele; D, homozygous wildtype frequency (the frequency of reference allele homozygote); H, heterozygous mutant frequency; HWE, Hardy Weinberg Equilibrium; R, homozygous mutant frequency (homozygote frequency for the other allele).

**TABLE 3:** Haplotypes of SNPs loci sites of TGFβR1 and TGFβRII

Gene	CHR	SNPs Loci	Haplotype name	Haplotype	Haplotype frequency	Estimate	*p*‐Value
*TGFβR1*	2	g.9414A > G g.28881A > G g.28809T > C g.10429G > A	H1	AGCA	0.3300	0.29512	0.31867
			H2	AGTA	0.6994	–	–
			H3	GACG	0.0676	−0.3428	0.63107
*TGFβRII*	19	g.63940C > T g.63976C > T	H4	CC	0.690	–	–
			H5	CT	0.1437	−0.0069	0.98795
			H6	TC	0.3763	0.53994	0.04208

**TABLE 4:** Population genetic structure of *TGFβR1* and *TGFβRII* gene

Gene	Loci	Gene homozygosity (Ho)	Gene heterozygosity (He)	Effective allele numbers (Ne)	Polymorphic information content
*TGFβR1*	g.9414A > G	0.88	0.12	1.13	0.11
	g.28881A > G	0.88	0.12	1.13	0.11
	g.28809T > C	0.52	0.48	1.91	0.36
	g.10429G > A	0.88	0.12	1.13	0.11
*TGFβRII*	g.63940C > T	0.53	0.47	1.89	0.36
	g.63976C > T	0.79	0.21	1.27	0.19
	g.64538C > T	0.93	0.07	1.08	0.07
	g.64504T > A	0.92	0.08	1.09	0.08

### 3.4 Association analysis of SNPs with litter size in Tibetan sheep

The effects of Tibetan sheep *TGFβRI* and *TGFβRII* SNPs on litter size of the experimental populations were studied. The results showed that the g.9414A > G, g.28881A > G, g.28809T > C, and g.10429G > A of sheep *TGFβRI* were associated with litter size (*p* < 0.05). In contrast, the *TGFβRII* g.63940C>T substitution was associated with litter size (*p* < 0.05). However, the SNPs, g.63976C > T, g.64538C > T, and g.64538C > T had no association with litter size (Table 5). All results indicated that *TGFβRI* and *TGFβRII* contributed to phenotype values.

**TABLE 5:** The correlation of litter size and genotypes of *TGFβR1* and *TGFβRII* gene

Gene	Loci	Genotype	Number	Litter size
*TGFβR1*	g.9414A > G	AA	383	1.07 ± 0.26^b^
		AG	45	1.00 ± 0.00^b^
		GG	5	1.40 ± 0.55^a^
	g.28881A > G	AA	383	1.07 ± 0.26^b^
		AG	45	1.00 ± 0.00^b^
		GG	5	1.40 ± 0.55^a^
	g.28809T > C	TT	154	1.09 ± 0.29^b^
		TC	217	1.03 ± 0.16^b^
		CC	62	1.16 ± 0.37^a^
	g.10429G > A	GG	412	1.07 ± 0.26^b^
		GA	44	1.00 ± 0.00^b^
		AA	7	1.40 ± 0.55^a^
*TGFβRII*	g.63940C > T	CC	175	1.05 ± 0.21^b^
		CT	188	1.07 ± 0.26^ab^
		TT	70	1.11 ± 0.32^a^
	g.63976C > T	CC	336	1.07 ± 0.26
		CT	90	1.07 ± 0.25
		TT	7	1.00 ± 0.00
	g.64538C > T	CC	402	1.07 ± 0.25
		CT	29	1.14 ± 0.35
		TT	2	–
	g.64504T > A	TT	395	1.07 ± 0.25
		TA	38	1.11 ± 0.31
		AA	0	–

*Note*: Least squares means with the same superscript have no significant difference (*p* > 0.05). Least squares means with the different superscripts differ significantly (*p* < 0.05).

## 4 DISCUSSION

TGFβ superfamily is evolutionarily conserved and plays fundamental roles in cell growth and differentiation (Attisano & Wrana, 1996; Hill, 1996). TGFβ superfamily signalling is essential for female reproduction (Li, 2014), and TGFβ superfamily affects the reproductive physiology of animals (Nie et al., 2014), for example, influencing the development of follicles by regulating the proliferation or apoptosis of Granulosa cells in the follicles and causing follicular atresia (Li, 2014; Nie et al., 2014; Ovchinnikov & Wolvetang, 2011). TGFβRI and TGFβRII, core components of TGF‐β superfamily, are important intraovarian growth factors (Ester et al., 1999), so *TGFβRI* and *TGFβRII* genes were used as candidate genes for reproductive traits to study. TGFβRI and TGFβRII are serine‐threonine kinases that signal through the Smad family of proteins (Ovchinnikov & Wolvetang, 2011; Sun et al., 2008). TGFβ1 binds to the TGFβRII, which in turn recruits the binding of TGFβRI to form a heterotetramer. TGFβRI then phosphorylates and activates the Smad2 protein (Li, 2014; Nie et al., 2014) after combining with Smad4, followed by translocation to the nucleus where the activated Smad complex. Then, it is involved in regulating transcriptional responses on target genes (Ikushima & Miyazono, 2010). At present, there are few studies on the structural characterization of *TGFβRI* and *TGFβRII*. In this study, we analyzed the homology of sheep TGFβRI and TGFβRII proteins with 10 other species, respectively. It was found that TGFβRI and TGFβRII have a higher percentage of sequences homology indicating that TGFβRI and TGFβRII were conserved across the above‐mentioned species.

Type I and type II TGFβ receptors appear to be ubiquitously expressed in most cell types (Knight & Glister, 2006). The tissue expression profiles revealed that *TGFβRI* and *TGFβRII* have broad expression patterns in Tibetan sheep. Ovarian cells have been shown to produce TGFβRI and TGFβRII, whose expression was first detected in preantral follicles and continues throughout the subsequent stages of follicular development (Knight & Glister, 2006). The mRNA and proteins of TGFβ receptors type I and II exist in the human oocyte, and receptor type I exists in blastocysts, indicating a selective expression of transcripts for TGFβ receptors in oocytes and blastocysts (Osterlund & Fried, 2000). Expression of TGFβRI mRNA was observed in the sheep ovary, while expression of TGFβRII mRNA within the follicle was limited to the theca (Juengel et al., 2004). The expression of TGFβR mRNA/protein in preantral follicles has been documented in several species including rodents, human, sheep, and cattle (Chow et al., 2001; Juengel et al., 2004; Osterlund & Fried, 2000; Roy, 2000). We found that both *TGFβRI* and *TGFβRII* were expressed in ovary, oviduct, uterus, hypothalamus, and hypophysis, as well as in other tissues. We also found that expression of *TGFβRI* was the highest in lung, followed by spleen, uterus, and ovary, and *TGFβRII* was higher in uterus than in the other tissues.

TGFβRI and TGFβRII are essential for regulating the growth and differentiation of ovarian follicles and thus fertility (Juengel et al., 2004). Osterlund and Fried (2000) reported that TGFβ receptor types I and II are present in human oocytes. Juengel et al. (2004) reported that the expression of mRNAs encoding TGF‐β1 and TGF‐β2 as well as both type I and II TGF‐β receptors were observed in the theca of small growing follicles indicating that TGF‐βs may be regulating thecal cell function in an autocrine manner. Expression of mRNA encoding TGF‐β type I and II receptors was observed in luteal cells, stroma, the vascular system, and surface epithelium suggesting that TGF‐βs may also regulate other cell types in the sheep ovary (Juengel et al., 2004). A similar pattern of expression for the TGFβRII mRNA was observed in mouse follicles, with expression most prominent in the theca and barely detectable in granulosa cells (Juengel et al., 2004). TGFβRI and TGFβRII are important cell regulators that play important regulatory roles in ovary development and animal reproduction. In this study, g.9414A > G, g.28881A > G, g.28809T > C, g.10429G > A in *TGFβRI*, and g.63940C > T in *TGFβRII* were associated with litter sizes in Tibetan sheep, and *TGFβRI* and *TGFβRII* can be used as molecular markers for improving the reproduction performance of Tibetan sheep. However, further studies on the association between the two genes and productive performance of different sheep breeds are required.

## 5 CONCLUSIONS

In this study, we cloned cDNA sequences of *TGFβRI* and *TGFβRII* genes in Tibetan sheep and the sequences homology of the two genes was the most similar to *O. aries*, followed by *B. mutus*. We also found that *TGFβRI* and *TGFβRII* were expressed in the different tissues of Tibetan sheep, and the expression of *TGFβRI* was the highest in lung, followed by spleen, uterus, and ovary, and *TGFβRII* expression was higher in uterus than the other tissues. The g.9414A > G, g.28881A > G, g.28809T > C, g.10429G > A mutations of TGFβRI and g.63940C > T of TGFβRII were screened out, and three different genotypes as well as three different haplotypes were identified for each gene. The g.9414A > G, g.28881A > G, g.28809T > C, and g.10429G > A mutation of sheep *TGFβRI* had an association with litter size, and the *TGFβRII* g.63940C > T was associated with litter size. Thus, our results indicate that *TGFβRI* and *TGFβRII* can be used as candidate genes for the improvement of reproductive performance of Tibetan sheep during breeding.

## AUTHOR CONTRIBUTIONS

*Formal analysis, methodology, validation, and writing—original draft, and writing—review and editing*: Junxia Zhang. *Data curation and investigation*: Mingming Li, Na He, and Ruizhe Sun: *Conceptualization, methodology, and writing—review and editing*: Xiaocheng Wen. *Data curation and validation*: Xueping Han. *Conceptualization, methodology, and writing—review and editing*: Zenghai Luo.

## CONFLICTS OF INTEREST

The authors declare no conflict of interest.

## FUNDING INFORMATION

The funds of Science and Technology Planning Program of Qinghai (Science and Technology Department of Qinghai Province) (Grant No. 2020‐ZJ‐786); Outstanding Person of Kunlong: Rural Revitalization Program (Grant No. (2020)9).

### ETHICS STATEMENT

All experiments in this study were performed following the approved guidelines of the Regulation of the Standing Committee of Qinghai People's Congress. All experimental protocols and the collection of samples were approved by the Ethics Committee of Qinghai University under permission No. SL‐2021027.

### PEER REVIEW

The peer review history for this article is available at https://publons.com/publon/10.1002/vms3.1013.

## Data Availability

The data that support the findings of this study will be shared upon reasonable request to the corresponding author.
